# A Mini-Review: Fabrication of Polysaccharide Composite Materials Based on Self-Assembled Chitin Nanofibers

**DOI:** 10.3390/ma17081898

**Published:** 2024-04-19

**Authors:** Jun-ichi Kadokawa

**Affiliations:** Graduate School of Science and Engineering, Kagoshima University, 1-21-40 Korimoto, Kagoshima 890-0065, Japan; kadokawa@eng.kagoshima-u.ac.jp; Tel.: +81-99-285-7743

**Keywords:** chitin nanofiber, composite, physical interaction, polysaccharide, self-assembly

## Abstract

This mini-review presents the fabrication methods for polysaccharide composite materials that employ self-assembled chitin nanofibers (ChNFs) as functional components. Chitin is one of the most abundant polysaccharides in nature. However, it is mostly not utilized because of its poor feasibility and processability. Self-assembled ChNFs are efficiently obtained by a regenerative bottom-up process from chitin ion gels using an ionic liquid, 1-allyl-3-methylimodazolium bromide. This is accomplished by immersing the gels in methanol. The resulting dispersion is subjected to filtration to isolate the regenerated materials, producing ChNF films with a morphology defined by highly entangled nanofibers. The bundles are disintegrated by electrostatic repulsion among the amino groups on the ChNFs in aqueous acetic acid to produce thinner fibers known as scaled-down ChNFs. The self-assembled and scaled-down ChNFs are combined with other chitin components to fabricate chitin-based composite materials. ChNF-based composite materials are fabricated through combination with other polysaccharides.

## 1. Introduction

**Figure 1:**
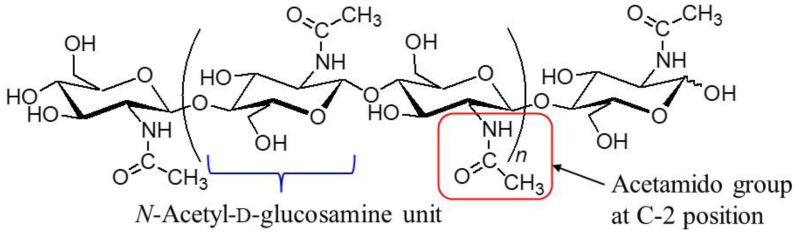
Chemical structure of chitin.

The production of value-added bio-based materials through biomass conversion has attracted considerable attention as an alternative to fossil-resource-based materials [1]. After cellulose, chitin is the second most abundant biomass and is mainly biosynthesized in the exoskeletons of crustaceans such as shrimps and crabs [2,3,4,5]. Due to its extended chain structure consisting of β(1→4)-linked *N*-acetyl-d-glucosamine repeating units, chitin has a highly fibrous crystallinity and stiff molecular chain packing, which is primarily constructed through the strong intermolecular hydrogen bonding between the acetamido groups at the C-2 position (Figure 1). Therefore, chitin exhibits poor solubility in common solvents, which leads to difficult processability and utility as a material. Accordingly, the conversion of chitin into value-added materials has attracted increasing attention in research on chitin in recent years [6,7,8,9].

Due to their remarkable properties, including light weight, high tensile strength, and low thermal expansion coefficients, nanofibrillated materials (e.g., nanofibers, nanocrystals, and nanowhiskers) have been widely identified as efficient functionalized polysaccharides [10]. Chitin nanomaterials exhibit additional properties for practical applications [11] such as biomedical applications [12], nanosheet formability for sensing and electronic devices [13], oral absorbents and administration [14,15], barrier applications [16], high-performance strong and tough films [17], and optical-sensing applications [18]. To produce these nanofibrillated chitins from native sources, two practical processes, that is, top-down and bottom-up approaches, are typically employed, which involve chitin microfibril disentanglement and self-assembly through regeneration from chitin solutions/gels, respectively [19].

**Figure 2:**
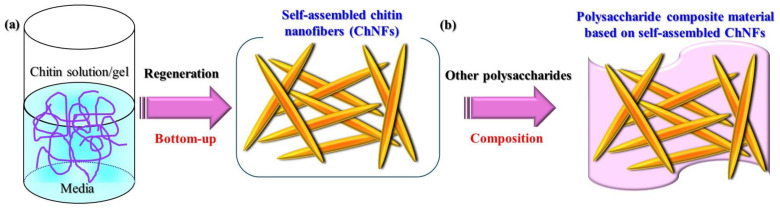
(**a**) Preparation of self-assembled chitin nanofibers (ChNFs) by regenerative bottom-up procedure and (**b**) fabrication of polysaccharide composite materials based on self-assembled ChNFs.

Because native chitin sources in crustacean shells are formed as microfibrils with an embedded protein matrix, which are constructed by the assembly of nanofibers with diameters of 2–5 nm [20,21], some top-down approaches, such as mechanical treatment by grinding, have been employed to fabricate chitin nanofibers (ChNFs) [22,23,24,25,26,27]. This author developed a facile top-down technique through the disentanglement of native chitin powder to obtain an aqueous dispersion of ChNFs, which was achieved by N_2_ gas bubbling and ultrasonic treatment in water [28]. Amino groups were generated on the resulting ChNFs by the partial *N*-deacetylation of chitin molecules (known as partially deacetylated (PDA)-ChNFs), which were efficiently converted to cationic (amidinium) groups through successive amidination and cationization with CO_2_ to construct cationic ChNFs. By contrast, suitably adjusted conditions for regeneration from chitin solutions/gels has resulted in self-assembled ChNFs according to the bottom-up procedure (Figure 2a) [29,30]. Electrospinning has also been used for the self-assembly of ChNFs from chitin solutions [31,32].

Furthermore, nanocomposite materials derived from polymeric components have endless applications, ranging from low-cost household products to high-value industrial production entities. For example, cellulose-based nanocomposites have recently attracted considerable interest [33] as they have suitable properties for use in various polymer nanocomposite preparations. These properties include low density, non-abrasiveness, combustibility, nontoxicity, and biodegradability. They are also less expensive than other synthetic polymers.

The composition of self-assembled ChNFs on other polysaccharide substrates has also been attempted for the production of ChNF-based composite materials (Figure 2b) [34]. This mini-review provides an overview of the fabrication of polysaccharide composite materials based on the aforementioned self-assembled ChNFs. The preparation procedures for self-assembled ChNFs and their further treatment into scaled-down fibrils (SD-ChNFs) are introduced. The compositions of the obtained ChNFs with other types of chitin substrates are then described in fabricating composite materials consisting solely of chitin components. The fabrication of composite materials from ChNFs and other polysaccharides is also described.

## 2. Preparation of Self-Assembled ChNFs from Ion Gels and Their Further Scaling Down into Thinner Fibrils

**Figure 3:**
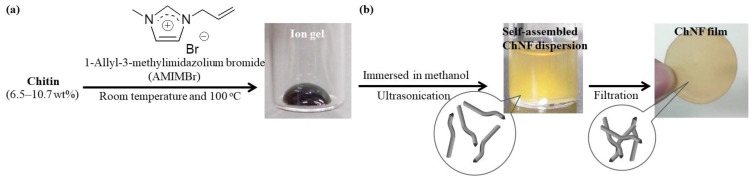
Procedures for formation of (**a**) chitin/1-allyl-3-3metnylimidazolium bromide (AMIMBr) ion gel and (**b**) self-assembled ChNFs (film).

Ionic liquids (ILs) have been employed as media for the preparation of ChNFs through a regenerative self-assembly process [30,35]. ILs are molten salts with melting points below the boiling temperature of water. ILs are known to be powerful solvents for natural polysaccharides (e.g., cellulose [36,37,38,39,40,41]). In 2002, an ionic liquid, 1-butyl-3-methylimidazolium chloride (BMIMCl), was found to dissolve cellulose [42]. Due to the poorer feasibility of chitin as compared with other polysaccharides, ILs that can dissolve chitin were hardly known until approximately 15 years ago [43,44,45,46,47]. Indeed, 1-Butyl-3-methylimidazolium acetate was first used for chitin dissolution in 2008 [48]. In 2009, this author found that the chitin dissolution ability of 1-allyl-3-methylimidazolium bromide (AMIMBr) was as high as 4.8 wt% [49]. In addition, ion gels were observed as forming from larger mixtures of chitin with AMIMBr (6.5–10.7 wt%) through successive holdings at room temperature and heating at 100 °C (Figure 3a).

When the chitin/AMIMBr ion gels were immersed in methanol with ultrasonication for regeneration, ChNFs of 20–60 nm in width and several hundred nm in length were fabricated through self-assembly at the nanoscale to form methanol dispersions (Figure 3b) [50,51]. The resulting self-assembled ChNFs were isolated via the filtration of the dispersions, leading to the formation of ChNF films with a highly entangled nanofiber morphology. The transmission electron microscopy measurements of the resulting ChNF/methanol dispersion revealed that the self-assembled ChNFs were constructed as bundle assemblies of thinner fibrils with average widths and lengths of 12 and 163 nm, respectively [52].

**Figure 4:**
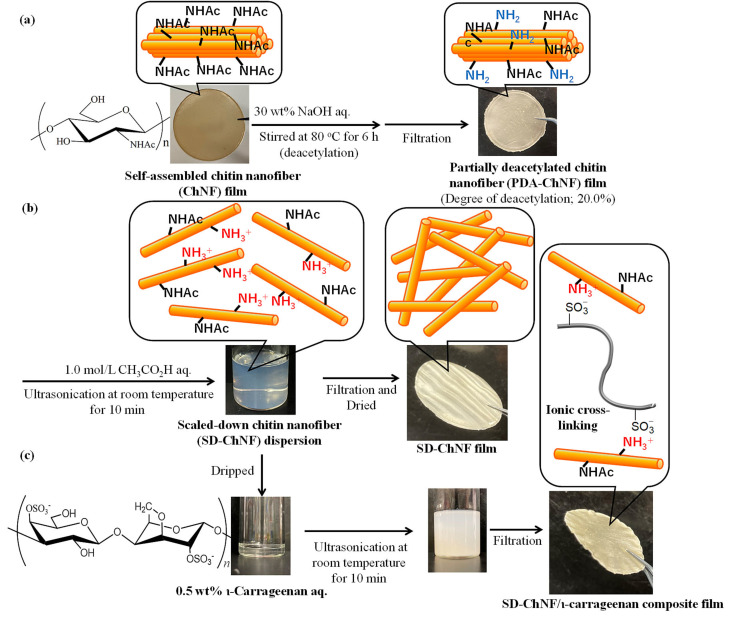
Procedures for (**a**) preparation of partially deacetylated chitin nanofiber (PDA-ChNF) film, (**b**) fabrication of scaled-down ChNF (SD-ChNF) film, and (**c**) composition of SD-ChNF with ι-carrageenan (adapted with permission from Ref. [53]. Copyright 2021, Elsevier).

For the disintegration of the bundles by electrostatic repulsion, amino groups were partly generated on the self-assembled ChNFs by the partial deacetylation of the acetamido groups via treatment with aqueous NaOH (Figure 4a). The obtained PDA-ChNFs were then mixed with aqueous acetic acid (1.0 mol/L) under ultrasonication to yield individual thin fibrils by electrostatic repulsion among the ammonium groups on the ChNFs and identified as SD-ChNFs (Figure 4b) [53]. The SD-ChNFs produced were isolated via the suction filtration of the dispersion, leading to the formation of a highly flexible film with a heavily entangled morphology from the thin fibrils. The author confirmed that this film formation occurred through gelation due to the concentration of the dispersions during the suction filtration.

## 3. Composition of Self-Assembled ChNFs and SD-ChNFs with Other Chitin Components

**Figure 5:**
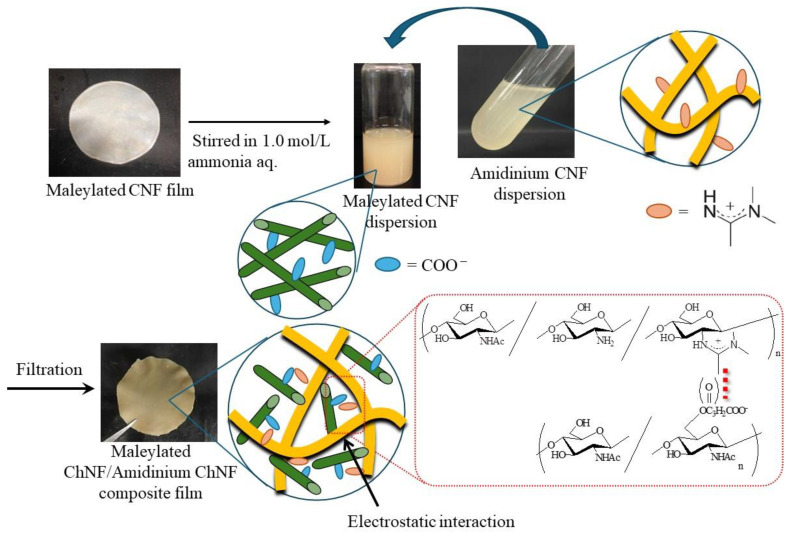
Procedure for preparation of composite film from cationic/anionic chitins (reprinted with permission from Ref. [54]. Copyright 2018, Springer).

To fabricate composite materials from the aforementioned cationic ChNFs with amidinium groups by electrostatic interaction, anionic groups were introduced on the self-assembled ChNFs. Accordingly, the reaction of the hydroxy groups on the self-assembled ChNF film with maleic anhydride was performed using perchloric acid as an acid catalyst to introduce carboxylate groups [54]. The scanning electron microscopy (SEM) image of the product shows that it retained its nanofiber morphology. Because the obtained anionic ChNF film was well-dispersed in aqueous ammonia (1.0 mol/L), this dispersion was mixed with the aqueous cationic CNF dispersion to improve the composition via electrostatic interaction (Figure 5). The obtained dispersion that included the two chitin components was filtered to produce composite films [54]. The tensile testing of the products suggested that the mechanical properties were enhanced based on the degree of substitution of the cationic and anionic groups on the ChNFs and when the molar ratio of these groups approached 1:1.

**Figure 6:**
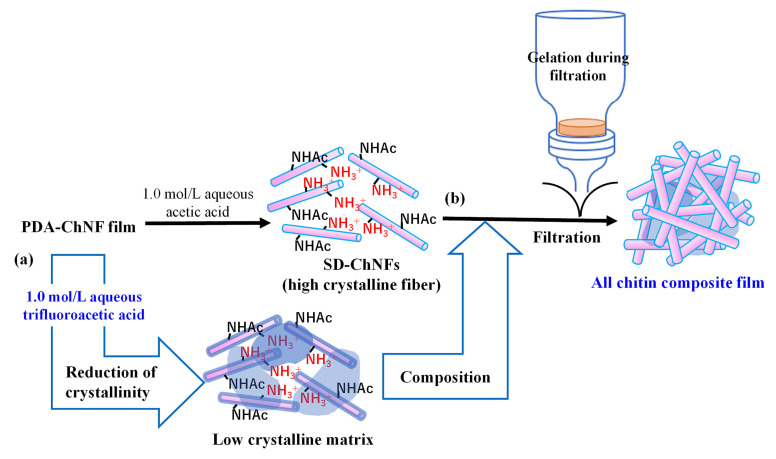
Procedures for preparation of (**a**) low-crystalline chitin matrix dispersion with 1.0 mol/L aqueous trifluoroacetic acid and (**b**) all-chitin composite film.

Composite materials based solely on polymers are referred to as all-polymer composites [55,56]. An all-cellulose composite, which is one of the most extensively studied all-polymer composites, is fabricated solely from cellulose, which acts as both an incorporated fiber reinforcement with high crystallinity and a matrix with low crystallinity [57,58,59]. By contrast, the fabrication of all-chitin composites has not been reported thus far because of the difficulty in obtaining low-crystalline components. This author exploited the gelation process during the filtration from the aforementioned SD-ChNF/aqueous acetic acid dispersion to obtain chitin matrices with low crystallinity. The crystallinity was reduced by treatment with aqueous trifluoroacetic acid (1.0 mol/L) at room temperature for 10 min via ultrasonication and at 50 °C for 24 h under stirring to produce a low-crystalline matrix dispersion (Figure 6a) [60]. The resulting dispersion was mixed with a highly crystalline SD-ChNF/aqueous acetic acid dispersion, followed by filtration through gelation to fabricate an all-chitin composite film (Figure 6b). Appropriate weight ratios of the two components in the all-chitin composite films resulted in superior mechanical properties in the tensile mode as compared with those of the sole SD-ChNF film. For example, the tensile strength values of the former composite (low-crystalline matrix: high-crystalline fiber = 0.026:1 (*w*/*w*)) and the latter sole films were 78.0 and 44.5 MPa, respectively. This study presents the first example of the fabrication of an all-chitin composite.

## 4. Composition of Self-Assembled ChNFs and SD-ChNFs with Other Polysaccharide Components

**Figure 7:**
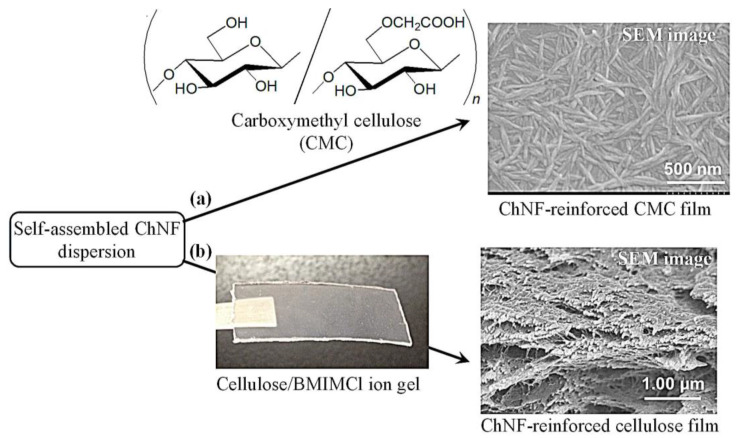
(**a**) Procedures for preparation of ChNF-reinforced (**a**) CMC film and (**b**) cellulose film.

Self-assembled ChNFs were employed as reinforcing agents in the fabrication of composites with cellulosic materials. For example, an anionic derivative, namely carboxymethyl cellulose (CMC), was identified by the self-assembled ChNFs through electrostatic interaction. Chitin is regarded as a cationic polysaccharide because of the presence of a high percentage of amino groups in the total repeating units by the deacetylation of the acetamido groups [61]. The CMC films formed using the casting method were immersed in different concentrations of self-assembled ChNF/methanol dispersions (Figure 7a). The centrifugation and drying of the mixtures yielded the ChNF-reinforced films. The presence of nanofibers on the surface was supported by the SEM image of the resulting film.

Self-assembled ChNF-reinforced cellulosic films were also prepared. The aforementioned ionic liquid, BMIMCl, has been reported to form a cellulosic ion gel [62]. Self-assembled ChNFs and cellulose were composited by immersing the cellulosic ion gels in self-assembled ChNF/methanol dispersions containing different amounts of chitin (Figure 7b) [63]. The mixtures were centrifuged to promote cellulose regeneration, resulting in self-assembled ChNF-reinforced cellulosic films. The ChNF/cellulose unit ratios in the films increased with increasing amounts of chitin in the methanol dispersion. The SEM images of the films indicated that the ChNFs were present not only on the surfaces but also inside the films as the ChNF tip morphology extending from the solid was observed in the cross-sectional area. The enhancement of the mechanical properties of the CMC and cellulose composite films under tensile mode was confirmed by increasing the amount of ChNFs in the film, supporting the reinforcing effect of the ChNFs.

**Figure 8:**
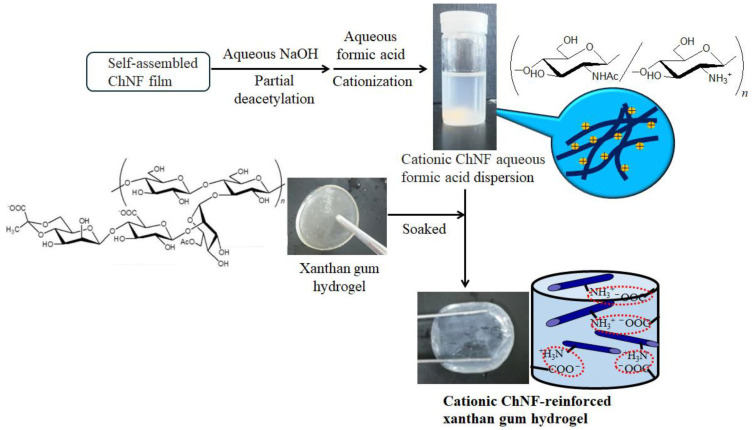
Procedure for preparation of ChNF-reinforced xanthan gum film (adapted with permission from Ref. [64]. Copyright 2020, Springer).

The self-assembled ChNFs were cationized and then employed as a reinforcing agent for a xanthan gum hydrogel, which is an anionic polysaccharide with carboxylate groups (Figure 8) [64]. The self-assembled ChNFs were first treated with aqueous NaOH for partial *N*-deacetylation. The amino groups of the generated ChNFs were then protonated using aqueous formic acid. Xanthan gum hydrogels were prepared from xanthan gum and BMIMCl based on a previously reported procedure [65]. The ion gel was first produced by heating–cooling a mixture of xanthan gum and BMIMCl. The ion gel was then immersed in water to exchange the dispersed media and obtain a xanthan gum hydrogel. The hydrogel was immersed in a cationic ChNF aqueous dispersion for composition via the ion exchange between ammonium formates and carboxylate salts. The degree of deacetylation strongly affected the amount of ChNFs in the resulting composite hydrogels. The compression testing of the hydrogels was conducted to evaluate the reinforcing effect of the ChNFs, which were strengthened with increasing degrees of deacetylation. This effect was likely induced by the electrostatic interaction between the two polysaccharides.

The composition of the aforementioned SD-ChNFs with anionic ι-carrageenan and sulfate groups was attempted through multipoint ionic cross-linking. The SD-ChNFs/aqueous acetic acid dispersion was dripped on a viscous 0.5 wt% aqueous ι-carrageenan, which was then filtered to obtain a composite film (Figure 4c) [53].

**Figure 9:**
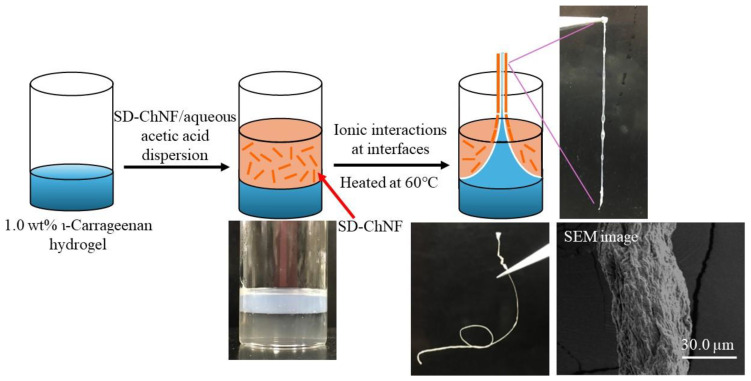
Procedure for preparation of ChNF/ι-carrageenan composite fiber.

When the SD-ChNFs/aqueous acetic acid dispersion was gently placed on a 1.0 wt% ι-carrageenan hydrogel, the two-layer system was obtained. After the media were heated at 60 °C for the fluidity of the hydrogel, the solidified product at the interfacial area was continuously extracted to produce a fibrous material and then dried to obtain the flexible and knottable fiber (Figure 9). The SEM images of the resulting fiber revealed an entangled nanofiber morphology, which was probably formed under the multi-point ionic cross-linking of the SD-ChNFs with ι-carrageenan.

## 5. Conclusions

This mini-review provided an overview of the fabrication of polysaccharide composite materials based on self-assembled ChNFs. Studies on the fabrication methods of ChNF-based composite materials have been conducted over the last 15 years and will continue to attract significant attention in application fields related to the environmental and biomedical industries in the future. Composite materials from SD-ChNFs were also reviewed in this mini-review. These ChNFs were composited with other chitin and polysaccharide components, such as cellulose, xanthan gum, and ι-carrageenan. Further studies on developing new preparation methods for ChNFs with different sizes and morphologies will be conducted using a bottom-up approach to provide additional polysaccharide composite materials with suitable properties and applications.

## Data Availability

Not applicable.

## References

[1] Daramola M.O., Ayeni A.O. (2020). Valorization of Biomass to Value-Added Commodities: Current Trends, Challenges, and Future Prospects.

[2] Rinaudo M. (2006). Chitin and chitosan: Properties and applications. Prog. Polym. Sci..

[3] Kurita K. (2006). Chitin and chitosan: Functional biopolymers from marine crustaceans. Mar. Biotechnol..

[4] Pillai C.K.S., Paul W., Sharma C.P. (2009). Chitin and chitosan polymers: Chemistry, solubility and fiber formation. Prog. Polym. Sci..

[5] Iber B.T., Kasan N.A., Torsabo D., Omuwa J.W. (2022). A review of various sources of chitin and chitosan in nature. J. Renew. Mater..

[6] Rinaudo M. (2014). Materials based on chitin and chitosan. Bio-Based Plastics: Materials and Applications.

[7] Li B., Mu X. (2017). Recent progress in the utilization of chitin/chitosan for chemicals and materials. Fuels, Chemicals and Materials from the Oceans and Aquatic Sources.

[8] Maleki G., Milani J.M. (2020). Functional properties of chitin and chitosan-based polymer materials. Handbook of Chitin and Chitosan: Volume 3: Chitin- and Chitosan-Based Polymer Materials for Various Applications.

[9] Halloub A., Marya R., Essabir H., Bouhfid R., El Kacem Qaiss A. (2023). Chitin and chitosan derivatives to proffer new functional materials. Polysaccharides: Advanced Polymeric Materials.

[10] Anusiya G., Jaiganesh R. (2022). A review on fabrication methods of nanofibers and a special focus on application of cellulose nanofibers. Carbohydr. Polym. Technol. Appl..

[11] Iroegbu A.O.C., Ray S.S. (2023). Chitin nanomaterials as multifunctional systems in advanced applications—Progress and challenges toward sustainability. Macromol. Mater. Eng..

[12] Muzzarelli R.A.A., El Mehtedi M., Mattioli-Belmonte M. (2014). Emerging biomedical applications of nano-chitins and nano-chitosans obtained via advanced eco-friendly technologies from marine resources. Mar. Drugs.

[13] You J., Li M., Ding B., Wu X., Li C. (2017). Crab chitin-based 2D soft nanomaterials for fully biobased electric devices. Adv. Mater..

[14] Anraku M., Tabuchi R., Ifuku S., Nagae T., Iohara D., Tomida H., Uekama K., Maruyama T., Miyamura S., Hirayama F. (2017). An oral absorbent, surface-deacetylated chitin nano-fiber ameliorates renal injury and oxidative stress in 5/6 nephrectomized rats. Carbohydr. Polym..

[15] Koizumi R., Azuma K., Izawa H., Morimoto M., Ochi K., Tsuka T., Imagawa T., Osaki T., Ito N., Okamoto Y. (2017). Oral administration of surface-deacetylated chitin nanofibers and chitosan inhibit 5-fluorouracil-induced intestinal mucositis in mice. Int. J. Mol. Sci..

[16] Satam C.C., Irvin C.W., Lang A.W., Jallorina J.C.R., Shofner M.L., Reynolds J.R., Meredith J.C. (2018). Spray-coated multilayer cellulose nanocrystal—Chitin nanofiber films for barrier applications. ACS Sustain. Chem. Eng..

[17] Mushi N.E., Nishino T., Berglund L.A., Zhou Q. (2019). Strong and tough chitin film from α-chitin nanofibers prepared by high pressure homogenization and chitosan addition. ACS Sustain. Chem. Eng..

[18] Naghdi T., Golmohammadi H., Yousefi H., Hosseinifard M., Kostiv U., Horák D., Merkoçi A. (2020). Chitin nanofiber paper toward optical (bio)sensing applications. ACS Appl. Mater. Interfaces.

[19] Kadokawa J. (2013). Preparation and applications of chitin nanofibers/nanowhiskers. Biopolymer Nanocomposites.

[20] Raabe D., Romano P., Sachs C., Fabritius H., Al-Sawalmih A., Yi S.B., Servos G., Hartwig H.G. (2006). Microstructure and crystallographic texture of the chitin–protein network in the biological composite material of the exoskeleton of the lobster Homarus americanus. Mater. Sci. Eng. A.

[21] Chen P.-Y., Lin A.Y.-M., McKittrick J., Meyers M.A. (2008). Structure and mechanical properties of crab exoskeletons. Acta Biomater..

[22] Ifuku S., Nogi M., Abe K., Yoshioka M., Morimoto M., Saimoto H., Yano H. (2009). Preparation of chitin nanofibers with a uniform width as α-chitin from crab shells. Biomacromolecules.

[23] Ifuku S., Nogi M., Yoshioka M., Morimoto M., Yano H., Saimoto H. (2010). Fibrillation of dried chitin into 10-20 nm nanofibers by a simple grinding method under acidic conditions. Carbohydr. Polym..

[24] Ifuku S., Saimoto H. (2012). Chitin nanofibers: Preparations, modifications, and applications. Nanoscale.

[25] Ifuku S. (2014). Chitin and chitosan nanofibers: Preparation and chemical modifications. Molecules.

[26] Ifuku S. (2015). Chitin nanofibers: Preparations, modifications, and applications. Handbook of Polymer Nanocomposites. Processing, Performance and Application: Volume C: Polymer Nanocomposites of Cellulose Nanoparticles.

[27] Ifuku S., Anraku M., Azuma K. (2021). Preparation of chitin nanofiber and its derivatives from crab shell and their efficient biological properties. Adv. Polym. Sci..

[28] Tanaka K., Yamamoto K., Kadokawa J. (2014). Facile nanofibrillation of chitin derivatives by gas bubbling and ultrasonic treatments in water. Carbohydr. Res..

[29] Rolandi M., Rolandi R. (2014). Self-assembled chitin nanofibers and applications. Adv. Colloid Interface Sci..

[30] Kadokawa J. (2015). Fabrication of nanostructured and microstructured chitin materials through gelation with suitable dispersion media. RSC Adv..

[31] Jayakumar R., Prabaharan M., Nair S.V., Tamura H. (2010). Novel chitin and chitosan nanofibers in biomedical applications. Biotechnol. Adv..

[32] Ganesh S.S., Anushikaa R., Swetha Victoria V.S., Lavanya K., Shanmugavadivu A., Selvamurugan N. (2023). Recent advancements in electrospun chitin and chitosan nanofibers for bone tissue engineering applications. J. Funct. Biomater..

[33] Sharma A., Thakur M., Bhattacharya M., Mandal T., Goswami S. (2019). Commercial application of cellulose nano-composites—A review. Biotechnol. Rep..

[34] Kadokawa J. (2022). Application of ionic liquids for the functional materialization of chitin. Mater. Adv..

[35] Kadokawa J. (2020). Processing techniques of chitin-based gels, blends, and composites using ionic liquids. Handbook of Chitin and Chitosan: Volume 2: Composites and Nanocomposites from Chitin and Chitosan, Manufacturing and Characterisations.

[36] Pinkert A., Marsh K.N., Pang S.S., Staiger M.P. (2009). Ionic liquids and their interaction with cellulose. Chem. Rev..

[37] Zakrzewska M.E., Bogel-Łukasik E., Bogel-Łukasik R. (2010). Solubility of carbohydrates in ionic liquids. Energy Fuels.

[38] Gericke M., Fardim P., Heinze T. (2012). Ionic liquids—Promising but challenging solvents for homogeneous derivatization of cellulose. Molecules.

[39] Isik M., Sardon H., Mecerreyes D. (2014). Ionic liquids and cellulose: Dissolution, chemical modification and preparation of new cellulosic materials. Int. J. Mol. Sci..

[40] Li Y., Wang J., Liu X., Zhang S. (2018). Towards a molecular understanding of cellulose dissolution in ionic liquids: Anion/cation effect, synergistic mechanism and physicochemical aspects. Chem. Sci..

[41] Bhat A.H., Khan I., Usmani M.A., Umapathi R., Al-Kindy S.M.Z. (2019). Cellulose an ageless renewable green nanomaterial for medical applications: An overview of ionic liquids in extraction, separation and dissolution of cellulose. Int. J. Biol. Macromol..

[42] Swatloski R.P., Spear S.K., Holbrey J.D., Rogers R.D. (2002). Dissolution of cellose with ionic liquids. J. Am. Chem. Soc..

[43] Wang W.T., Zhu J., Wang X.L., Huang Y., Wang Y.Z. (2010). Dissolution behavior of chitin in ionic liquids. J. Macromol. Sci. Phys..

[44] Jaworska M.M., Kozlecki T., Gorak A. (2012). Review of the application of ionic liquids as solvents for chitin. J. Polym. Eng..

[45] Kadokawa J. (2013). Ionic liquid as useful media for dissolution, derivatization, and nanomaterial processing of chitin. Green Sustain. Chem..

[46] Silva S.S., Mano J.F., Reis R.L. (2017). Ionic liquids in the processing and chemical modification of chitin and chitosan for biomedical applications. Green Chem..

[47] Shamshina J.L. (2019). Chitin in ionic liquids: Historical insights into the polymer’s dissolution and isolation. A review. Green Chem..

[48] Wu Y., Sasaki T., Irie S., Sakurai K. (2008). A novel biomass-ionic liquid platform for the utilization of native chitin. Polymer.

[49] Prasad K., Murakami M., Kaneko Y., Takada A., Nakamura Y., Kadokawa J. (2009). Weak gel of chitin with ionic liquid, 1-allyl-3-methylimidazolium bromide. Int. J. Biol. Macromol..

[50] Kadokawa J., Takegawa A., Mine S., Prasad K. (2011). Preparation of chitin nanowhiskers using an ionic liquid and their composite materials with poly(vinyl alcohol). Carbohydr. Polym..

[51] Tajiri R., Setoguchi T., Wakizono S., Yamamoto K., Kadokawa J. (2013). Preparation of self-assembled chitin nanofibers by regeneration from ion gels using calcium halide · dihydrate/methanol solutions. J. Biobased Mater. Bioener..

[52] Kadokawa J., Kawano A., Yamamoto K. (2019). Fabrication of semi-crystalline film by hexanoylation on self-assembled chitin nanofibers. ChemistrySelect.

[53] Hashiguchi T., Yamamoto K., Kadokawa J. (2021). Fabrication of highly flexible nanochitin film and its composite film with anionic polysaccharide. Carbohydr. Polym..

[54] Sato K., Yamamoto K., Kadokawa J. (2018). Preparation of cationic/anionic chitin nanofiber composite materials. J. Polym. Environ..

[55] Krajenta J., Safandowska M., Pawlak A., Galeski A. (2020). All-polymer composites—A new approach with the use of disentangled semi-crystalline polymers Part I. Disentangling and properties of disentangled polylactide. Polimery/Polymers.

[56] Krajenta J., Pawlak A., Galeski A. (2020). All-polymer composites—A new approach with the use of disentangled semi-crystalline polymers Part II. Preparation of composites from partially disentangled polylactide. Polimery/Polymers.

[57] Huber T., Müssig J., Curnow O., Pang S., Bickerton S., Staiger M.P. (2012). A critical review of all-cellulose composites. J. Mater. Sci..

[58] Baghaei B., Skrifvars M. (2020). All-Cellulose Composites: A Review of Recent Studies on Structure, Properties and Applications. Molecules.

[59] Uusi-Tarkka E.K., Skrifvars M., Haapala A. (2021). Fabricating sustainable all-cellulose composites. Appl. Sci..

[60] Egi Y., Kontani A., Kadokawa J. (2023). Fabrication of all-chitin composite films. Int. J. Biol. Macromol..

[61] Hatanaka D., Yamamoto K., Kadokawa J. (2014). Preparation of chitin nanofiber-reinforced carboxymethyl cellulose films. Int. J. Biol. Macromol..

[62] Kadokawa J., Murakami M., Kaneko Y. (2008). A facile preparation of gel materials from a solution of cellulose in ionic liquid. Carbohydr. Res..

[63] Kadokawa J., Endo R., Hatanaka D., Yamamoto K. (2015). Preparation of chitin nanofiber-reinforced cellulose films through stepwise regenerations from individually prepared ion gels. J. Polym. Environ..

[64] Kadokawa J., Noguchi S., Gotanda T., Kawano A., Yamamoto K. (2020). Fabrication of cationized chitin nanofiber-reinforced xanthan gum hydrogels. Polym. Bull..

[65] Izawa H., Kadokawa J. (2010). Preparation and characterizations of functional ionic liquid-gel and hydrogel materials of xanthan gum. J. Mater. Chem..

